# Editorial: Fruit detection and yield prediction on woody crops using data from unmanned aerial vehicles

**DOI:** 10.3389/fpls.2022.1112445

**Published:** 2022-12-13

**Authors:** Jorge Torres-Sánchez, Jefferson Souza, Salvatore Filippo di Gennaro, Francisco Javier Mesas-Carrascosa

**Affiliations:** ^1^ imaPing Group, Institute for Sustainable Agriculture (IAS-CSIC), Cordoba, Spain; ^2^ Faculty of Computing, Federal University of Uberlandia, Uberlandia, Brazil; ^3^ Institute for Bioeconomy, Department of Biology, Agriculture and Food Sciences, National Research Council (CNR), Sesto Fiorentino, Italy; ^4^ 4Department of Graphic Engineering and Geomatics, University of Cordoba, Cordoba, Spain

**Keywords:** UAV, precision agriculture (PA), machine learning - ML, deep learning, image analysis

Yield prediction is a significant factor in optimizing orchard management and can help to improve fruit quality in woody crops. Having accurate and timely yield predictions is required for making harvest and market plans. It provides relevant information about orchard variability necessary for planning fertilization, pruning, and water management in future seasons. Traditional yield prediction is based on manual, labor-intensive, and time-consuming measurements. Furthermore, these conventional estimations tend to be subjective and prone to errors.

Remote sensing has demonstrated its usefulness in agriculture, providing information from whole orchards overcoming the limits of manual scouting in various applications, from fertilizer prescription to pest detection or irrigation management. One of the best-suited remote sensing platforms for agriculture is the Unmanned Aerial Vehicle (UAV) because of their relatively low cost, the possibility of carrying a wide range of sensors, the ability to fly under the clouds, and the high spatial and temporal resolution of the data acquired by their sensors.

In recent years, advances in Artificial Intelligence (AI) have made significant achievements in object detection in images and 3D models. Applying such advances to images and 3D models generated from RGB cameras, multispectral sensors, and LiDAR sensors onboard ground platforms has proved helpful in fruit detection and yield prediction in woody crops. However, UAVs are usually more efficient than ground platforms in acquiring information from a whole orchard. Consequently, applying AI and other analysis techniques to data obtained from sensors onboard UAVs has potential in fruit detection and yield prediction, as some pioneering works have demonstrated. Therefore, the present Research Topic arose to contribute to the emerging field of yield prediction in woody crops using UAVs.

Among the works submitted to the Research Topic, we have selected three original contributions and one review article. Maheswari et al. reviewed deep learning-based segmentation techniques for fruit yield estimation in the context of smart farming. This work can serve the readers as a complete introduction to these techniques. The paper presented a detailed review of the steps involved in fruit detection, such as field sampling, data collection, annotation and augmentation, and model performance evaluation. Furthermore, the authors included a detailed analysis of the challenges in each one of these steps, exposing knowledge gaps that could be addressed in future research.

Although yield prediction in woody crops is usually related to estimating the number and weight of fruits, woody species also have edible leaves. In this context, Wu et al. focused their work on detecting the young edible leaves of *Toona sinensis*. They used hyperspectral imagery and trained five machine learning classification models using raw data and data preprocessed with six different methods. The Support Vector Machine model applied to the raw data presented the most reliable and robust prediction results, leading to the development of a fast and effective model for identifying the edible leaves of *Toona sinensis*.

The light intercepted by the canopy is a crucial variable for quantifying crop biomass development and yield potential. For this reason, Zhang et al. developed a method to predict almond yield using the estimated fractional photosynthetically active radiation (fPAR) as input variables. They used 3D models of the orchard created by UAV photogrammetry to evaluate fPAR and used this variable to develop regression models for almond yield prediction. The authors found that UAV-based estimation of fPAR was a strong yield indicator for ‘Nonpareil’ almond variety. The results of this article serve as a fundamental link between UAV estimations of fPAR and the actual yield of almonds.

Lin et al. presented a work for estimating the flowering rate of litchi trees because of the direct relationship between this variable and litchi yield. The authors trained a deep learning model based on YOLO architecture to detect flower clusters and flushes using RGB imagery acquired with a UAV. After detecting the flowers on the upper part of the litchi crowns, the authors fitted a model for estimating the total number of flowers in the whole crown. Results showed low error rates in the estimation of the flowering rate, demonstrating that the developed method can be used for flowering management, with influence on the fruit production of litchi trees.

The contributions to this Research Topic represent the wide range of methods used for yield prediction in woody crops using UAVs. They include different estimation methods, from classic linear models to machine learning and deep learning. There are examples of yield prediction using direct methodologies, like detecting edible leaves, and examples of the use of predicting variables such as flowering or the fPAR. Moreover, the selected articles also cover the use of different inputs, from RGB images to 3D models and hyperspectral imagery. In summary, we believe that the present Research Topic can be helpful for the readers as an introduction to the plethora of methods and sensors that can be used in yield prediction of woody crops using UAV data.

## Author contributions

All authors listed have made a substantial, direct and intellectual contribution to the work, and approved it for publication.

